# Maternally and Paternally Silenced Imprinted Genes Differ in Their Intron Content

**DOI:** 10.1002/cfg.437

**Published:** 2004-12

**Authors:** Marie E. Fahey, Walter Mills, Desmond G. Higgins, Tom Moore

**Affiliations:** 1 Department of Biochemistry Biosciences Institute University College Cork College Road Cork Ireland; 2 Department of Genetics University of Cambridge Downing Street Cambridge United Kingdom; 3 Bioinformatics Laboratory Conway Institute University College Dublin Belfield Dublin 4 Ireland

## Abstract

Imprinted genes exhibit silencing of one of the parental alleles during embryonic development. In a previous study imprinted genes were found to have reduced intron content relative to a non-imprinted control set (Hurst *et al*., 1996). However, due to the small sample size, it was not possible to analyse the source of this effect. Here, we re-investigate this observation using larger datasets of imprinted and control (non-imprinted) genes that allow us to consider mouse and human, and maternally and paternally silenced, imprinted genes separately. We find that, in the human and mouse, there is reduced intron content in the maternally silenced imprinted genes relative to a non-imprinted control set. Among imprinted genes, a strong bias is also observed in the distribution of intronless genes, which are found exclusively in the maternally silenced dataset. The paternally silenced dataset in the human is not different to the control set; however, the mouse paternally silenced dataset has more introns than the control group. A direct comparison of mouse maternally and paternally silenced imprinted gene datasets shows that they differ significantly with respect to a variety of intron-related parameters. We discuss a variety of possible explanations for our observations.


**Comparative and Functional Genomics**
*Comp Funct Genom* 2004; **5**: 572–583.
Published online in Wiley InterScience (www.interscience.wiley.com). **DOI:** 10.1002/cfg.437

**Research Paper**

### Maternally and paternally silenced imprinted genes differ in their intron content

Marie E Fahey[1#], Walter Mills[2], Desmond G Higgins[1#] and Tom Moore[1]*
[1]*Department of Biochemistry, Biosciences Institute, University College Cork, College Road, Cork, Ireland*
[2]*Department of Genetics, University of Cambridge, Downing Street, Cambridge, UK*

**Correspondence to:*
*Tom Moore, Department of Biochemistry, Biosciences Institute, University College Cork, College Road, Cork, Ireland.*
*E-mail: t.moore@ucc.ie*

[#]*Present address: Bioinformatics Laboratory, Conway Institute, University College Dublin, Belfield, Dublin 4, Ireland.*

Received: 2 September 2004
Revised: 1 November 2004
Accepted: 12 November 2004

### Abstract

**Imprinted genes exhibit silencing of one of the parental alleles during embryonic development. In a previous study imprinted genes were found to have reduced intron content relative to a non-imprinted control set (Hurst *et al.*, 1996). However, due to the small sample size, it was not possible to analyse the source of this effect. Here, we re-investigate this observation using larger datasets of imprinted and control (non-imprinted) genes that allow us to consider mouse and human, and maternally and paternally silenced, imprinted genes separately. We find that, in the human and mouse, there is reduced intron content in the maternally silenced imprinted genes relative to a non-imprinted control set. Among imprinted genes, a strong bias is also observed in the distribution of intronless genes, which are found exclusively in the maternally silenced dataset. The paternally silenced dataset in the human is not different to the control set; however, the mouse paternally silenced dataset has more introns than the control group. A direct comparison of mouse maternally and paternally silenced imprinted gene datasets shows that they differ significantly with respect to a variety of intron-related parameters. We discuss a variety of possible explanations for our observations. Copyright © 2005 John Wiley & Sons, Ltd.**

**Keywords:** **Genomic imprinting; intron evolution; intronless genes**

### Introduction

The intron content of a gene or genome is composed of the combined effects of intron length and intron number. Intron length varies greatly between species and between regions of a specific genome, e.g. the average length of an intron in the rat is greater than 1000 bp, whereas in the worm (*C. elegans*) it is less than 500 bp (Deutsch and Long, 1999). Within a specific genome, intron length is correlated with various genomic parameters, e.g. there is, on average, three times more intron DNA in regions of low, compared to high, GC content. This statistic varies depending on the species, ranging from 2.1 in the mouse to 4.0 in the ox (Duret *et al.*, 1995). Recombination rate (which is related to GC content) also correlates with intron length, with longer introns found in regions of low recombination (Carvalho and Clarke, 1999). In addition to genomic location, the functional characteristics of genes may influence intron content, e.g. highly expressed genes in the human have introns that are up to 14 times shorter than those found in genes expressed at a lower level, a finding that may be related to selection for increased transcriptional efficiency of highly expressed genes (Castillo-Davis *et al.*, 2002).

However, there is relatively little understanding of the factors that influence intron number. This is due largely to ignorance of the mechanisms by which new introns are generated in individual genomes, and the selective forces that govern their spread within, or removal from, the population (Rogozin *et al.*, 2003). Introns that have been fixed may evolve a variety of secondary functions associated with aspects of gene regulation, e.g. alternative splicing expands the repertoire

Copyright © 2005 John Wiley & Sons, Ltd.

of protein isoforms that can be produced from a single gene locus. However, new introns may initially impose a cost due to the occurrence of mutations that disrupt protein structure and function. Recently, Lynch took a population genetics approach and proposed a model of intron fixation in which the only relevant evolutionary forces considered were mutation and random genetic drift (Lynch, 2002). In this scheme, intron-containing alleles initially are subject to weak negative selection because they are more likely than intronless alleles to mutate to harmful variants, e.g. elimination of a splice recognition site may disrupt an open reading frame. If a species has a very large population size, e.g. prokaryotes, such a weak mutation pressure may be sufficient to prevent fixation of an intron. However, species with a low population size, such as large multicellular organisms, may allow fixation of an intron at the neutral rate, i.e. due to genetic drift. Such arguments may explain the increased 'intron load' observed in large organisms with small effective population sizes, relative to prokaryotes or simple eukaryotes (Lynch, 2002).

Genomic imprinting is a mechanism that causes monoallelic expression of a small number of genes with important functions during mammalian development (John and Surani, 1996). It has been proposed that the evolution of imprinting can be explained by the widespread occurrence of polyandry, which results in reduced relatedness of paternally, relative to maternally, derived alleles at embryonic loci that influence maternal investment, and can be understood as a form of intragenomic conflict (Moore and Haig, 1991). Previously, it was shown that, compared to a non-imprinted control set, imprinted genes have reduced intron content, having both fewer and shorter introns (Hurst *et al.*, 1996). This observation was interpreted in the context of the parental conflict theory as evidence for either selection to increase the transcription rate at imprinted loci (see subsequent work by Castillo-Davis *et al.*, 2002) or, more speculatively, to prevent degradation of mRNA due to putative mechanisms of 'splicing interference' (which would provide an additional negative selection pressure against intron fixation at imprinted loci; see Lynch, 2002). However, the small number of imprinted genes available to Hurst *et al.* (1996) did not allow firm conclusions regarding the source of the observed effect. We have assembled larger datasets of imprinted genes in both the mouse and human that allow us to examine their intron composition in detail.

### Results

### Control and imprinted gene datasets

Human and mouse Refseq datasets were used as non-imprinted controls (Pruitt and Maglott, 2001). To avoid subjective bias in imprinted gene selection, we used the imprinted gene list provided by the on-line *Catalogue of Imprinted Genes and Parent-of-Origin Effects Database* (hereafter called 'Human Imprinted Gene List') (Morison *et al.*, 2001; **http://cancer.otago.ac.nz/IGC/Web/home.html**) as an independent dataset of human imprinted genes. For the mouse, we used the online resource at the MRC Mammalian Genetics Unit (hereafter called 'Mouse Imprinted Gene List') (**http://www.mgu.har.mrc.ac.uk**). Genes with well-characterized intron structure were extracted from the lists to produce our human and mouse Modified Imprinted Gene Lists. We removed non-protein coding genes from the modified dataset and considered protein-coding imprinted genes separately because, apart from human *LIT1* and *H19*, and mouse *Igf2as, Peg13, Mirg, Air* and *H19*, many non-protein coding imprinted genes have poorly characterized gene structures. Also, the RefSeq control genes consist entirely of protein coding genes, and we cannot exclude a systematic difference in intron content between coding and non-coding genes. Human *IGF2as* was retained because a short open reading frame has been reported (Okutsu *et al.*, 2000). In addition, the following variations of the Modified Imprinted Gene Lists were also analysed:

1. Removal of genes with controversial or polymorphic imprinting status, i.e. human *GRB10* (Blagitko *et al.*, 2000), *IGF2R* (Kalscheuer *et al.*, 1993), *COPG2* (Yamasaki *et al.*, 2000).
2. Removal of intronless genes from both the RefSeq control and the Modified Imprinted Gene Lists.
3. Removal of *SNRPN* from the modified imprinted gene list as multiple small nucleolar RNA (snoRNA) molecules are encoded within some of its introns (Runte *et al.*, 2001). Clearly, the

Copyright © 2005 John Wiley & Sons, Ltd. 

evolutionary forces that influence the intronic structure of this gene are likely to be very different to those affecting other genes, particularly under the 'nearly neutral' model of intron evolution proposed by Lynch (2002).

### Maternally silenced imprinted genes have less intron content than non-imprinted control genes

We used the assembled datasets of human and mouse maternally and paternally silenced imprinted genes (Table 1) and compared them to human and mouse Refseq control datasets for a variety of parameters related to intron content, i.e. intron size and intron number. Using our Modified Imprinted Gene Lists, we find that both maternally and paternally silenced imprinted genes differ significantly from the control set with respect to intron content, but in different ways. Analysis of the human datasets, when non-protein coding genes are included, shows the total intron size of maternally silenced genes is significantly less than controls when *SNRPN* is removed (Table 2). Controlling for exon content, maternally silenced genes also contain significantly less intron sequence than the control dataset, when intronless genes are included. This finding is retained when non-coding genes and *SNRPN* are removed (Table 3). Removal of *SNRPN* also has a strong effect on average intron number.

Similar results were obtained for maternally silenced genes in the mouse; however, in general, they are less sensitive to *Snrpn* inclusion and significance is lost upon removal of intronless genes. Specifically, when controlled for exon content, maternally silenced genes have less intron content than controls. Also, average intron size is half that of the control set, but only when intronless genes are included. A similar trend of reduced intron size was observed in the human maternally silenced dataset, but was not statistically significant. Intron size co-varies with gene expression level (Castillo-Davis *et al.*, 2002). However, we found no difference between mouse or human maternally and paternally silenced imprinted datasets with respect to EST frequency in the public databases (mean EST count: human, paternally silenced, $162 \pm 92$; maternally silenced, $161 \pm 103$; Mann–Whitney, $p = 0.845$; mouse, paternally silenced, $92 \pm 87$; maternally silenced $135 \pm 99$; Mann–Whitney, $p = 0.07$).

### Mouse paternally silenced imprinted genes have higher intron content than non-imprinted control genes

Solely in the mouse, there are a significantly increased number of introns in the paternally silenced dataset, which has almost three times as many introns per kilobase of exon than the control set. This is a highly robust result, unaffected by removal of non-coding genes, intronless genes, *Snrpn* (Tables 2 and 3) or imprinted genes with controversial imprinting status. Also, total intron size and average intron size of mouse paternally silenced genes is higher than control genes, but this effect is lost when intronless genes are removed from the control dataset. A similar trend is observed in the human dataset, but fails to reach significance (Tables 3 and 4).

### Imprinted intronless genes are found exclusively in the maternally silenced dataset in the human and mouse

In the human and mouse, the maternally silenced gene datasets have a significantly higher proportion of intronless genes than the control dataset, whereas there are no intronless genes in the mouse and human paternally silenced datasets. Analyses were repeated following the removal of intronless genes from all datasets. Following this data manipulation, statistical difference with the control set was retained for the number of introns per kilobase of exon in the mouse paternally silenced dataset, and total intron size per kilobase of exon in the human maternally silenced dataset (Table 3). The majority of imprinted intronless genes are clustered at human chromosome 15q11–q13, and mouse chromosome 7, 28–29 cM (Table 1). There is a high degree of conservation of the mouse and human intronless imprinted gene orthologues with respect to chromosome map position, gene structure and imprinting status (Table 5). The exceptions are *Frat3*, which is a recent addition to the mouse Prader–Willi syndrome region adjacent to *Mkrn3* (Chai *et al.*, 2001), and *U2af1-rs1*, which is inserted in an intron of the biallelically expressed *Murr1* (Nabetani *et al.*, 1997). Inspection of Table 1 suggests that there is no correlation between the distribution of intronless genes and local recombination rate, neither is there an apparent correlation between maternal or paternal silencing

Copyright © 2005 John Wiley & Sons, Ltd. 

**Table 1.** Imprinted genes in human and mouse

| Human | | | Mouse | |
|---|---|---|---|---|
| Gene | Chromosome position | Recombination rate | Gene | Chromosome position |
| *NOEY2*[b] | 1p31 | 1.6 | *Gatm* | 2 E5 |
| *TP73* | 1p36.2 | 2.5 | ***Nnat*** | 2 88cM |
| ***ZAC1*** | 6q24–q25 | 0.5 | ***Gnasxl*** | 2 104cM |
| ***IGF2R***[c] | 6q26 | 0.6 | ***Nesp*** | 2 104cM |
| ***GRB10***[c] | 7p12–p11.2 | 1.2 | *Nespas* | 2 104cM |
| ***PEG10*** | 7q21 | 1.3 | *Sgce* | 6 1cM |
| ***DLX5*** | 7q22 | 1.0 | *Pon3* | 6 0.5cM |
| *CPA4* | 7q32 | 2.1 | *Pon2* | 6 A1 |
| ***COPG2***[c] | 7q32 | 2.1 | *Asb4* | 6 0.6cM |
| ***MEST*** | 7q32 | 2.1 | *Calcr* | 6 3.8cM |
| ***PEG1-AS*** | 7q32 | 2.1 | ***Peg10***[a] | 6 A1 |
| ***IGF2*** | 11p15.5 | 2.4 | ***Neurabin*** | 6 A1 |
| ***IGF2-AS*** | 11p15.5 | 2.4 | ***Mest*** | 6 7.5cM |
| ***INS*** | 11p15.5 | 2.4 | ***Copg2*** | 6 A3.3 |
| *LTRPC5* | 11p15.5 | 2.4 | ***Nap1l5*** | •6 |
| ***KVLQT1*** | 11p15.5 | 2.4 | *Zim1* | 7 6.5cM |
| ***CDKN1C*** | 11p15.5 | 2.4 | ***Peg3*** | 7 6.5cM |
| ***TSSC5*** | 11p15.5 | 2.4 | *Usp29* | 7 6.5cM |
| ***TSSC3*** | 11p15.5 | 2.4 | *Zim3* | 7 7.0cM |
| *ZNF215* | 11p15.4 | 1.7 | *Zfp264* | 7 7.0cM |
| *SDHD*[c] | 11q23 | 1.8 | ***Mkrn3*** | •7 28.0cM |
| *HTR2A* | 13q14–q21 | 1.0 | *Mkrn3–as*[a,b] | 7 |
| ***DLK1*** | 14q32 | 2.5 | ***Magel2*** | •7 28.0cM |
| ***MEG3*** | 14q32 | 2.5 | ***Ndn*** | •7 29.0cM |
| ***MKRN3*** | •15q11–q13 | 0.2 | *Frat3*[b] | •7 28.0cM |
| ***MAGEL2*** | •15q11–q12 | 0.2 | ***Ube3a*** | 7 28.65 |
| ***NDN*** | •15q11–q12 | 0.2 | ***Snrpn*** | 729.0cM |
| ***SNRPN*** | 15q12 | 2.5 | ***Igf2*** | 7 69.09cM |
| ***UBE3A*** | 15q11–q13 | 3.0 | ***Ins2*** | 7 69.1cM |
| *ATP10C* | 15q11–q13 | 3.0 | ***Ascl2*** | 7 69.3cM |
| ***PEG3*** | 19q13.4 | 0.4 | *Tssc4* | 7 69.3cM |
| ***NNAT*** | 20q11.2–q12 | 1.3 | ***Kcnq1*** | 7 F5 |
| ***GNASXL*** | 20q13.2–3 | 1.0 | ***Cdkn1c*** | 7 |
| ***NESP55*** | 20q13.2–3 | 1.0 | *Msuit*[a] | 7 69.49cM |
| **NESPAS** | 20q13.2–3 | | ***Tssc5*** | 7 69.5cM |
| | | | ***Tssc3*** | 7 69.5cM |
| | | | *Obph1* | 7 69.55cM |
| | | | *Nap1l4* | 7 69.59cM |

Copyright © 2005 John Wiley & Sons, Ltd. 

**Table 1.** Continued

| Human | | | Mouse | |
|---|---|---|---|---|
| Gene | Chromosome position | Recombination rate | Gene | Chromosome position |
| | | | *A19* | 7 69.59cM |
| | | | *Rasgrf1* | 9 50.0cM |
| | | | ***Zac1*** | 10 15.0cM |
| | | | *Dcn* | 10 55.0cM |
| | | | ***Grb10*** | 11 8.0cM |
| | | | ***U2af1-rs1*** | •11 12.0cM |
| | | | ***Dlk*** | 12 54.0cM |
| | | | ***Meg3*** | 12 54.5cM |
| | | | *Rian*[a] | 12 54.0cM |
| | | | *Dio3* | •12 F1 |
| | | | *Rtl1* | •12F1 |
| | | | ***Htr2a*** | 14 41.5cM |
| | | | *Ata3* | 15 F1 |
| | | | *Scl22a3* | 15F1 |
| | | | *Peg13* | •15 |
| | | | *Slc22a2* | 17 7.32cM |
| | | | ***Igf2r*** | 17 |
| | | | ***Air*** | •17 7.35cM |
| | | | *Impact* | 18 A2–B2 |
| | | | *Ins1* | 19 49.0cM |

Shaded boxes indicate conserved imprinted human/mouse orthologous gene pairs. Blue dots indicate intronless genes. Chromosomal location in blue indicates maternally silenced; in pink indicates paternally silenced. [a] Genes not included due to lack of, or insufficient, intron data. [b] No definitive mouse/human orthologue annotated. [c] Conflicting evidence of imprinting in the literature or exhibits polymorphic imprinting.

of intron-containing imprinted genes and chromosome map position or local recombination rate.

### Maternally and paternally silenced imprinted genes have different intron contents

To determine whether maternally and paternally silenced imprinted genes differ significantly from one another, the two datasets were compared directly for all parameters relating to intron content. None of the comparisons reached statistical significance in the human; however, in the mouse, all parameters, except those incorporating exon size, were significantly different (Table 6).

### Discussion

Using larger datasets, we have confirmed the finding of Hurst *et al.* (1996) that imprinted genes are unusual with respect to their intron content. Moreover, because of the relatively large number of new imprinted genes that were available for the current study, we were able to analyse mouse and human and maternally and paternally silenced datasets separately. Our major finding is that oppositely imprinted genes differ significantly in their intron content, and that this difference is directional: maternally silenced genes tend to have reduced intron content compared to controls, whereas paternally silenced genes tend to have increased intron content.

Copyright © 2005 John Wiley & Sons, Ltd. 

**Table 2.** Genomic structure of maternally and paternally silenced imprinted genes including non-protein coding genes

| | Human | | | Mouse | | |
|---|---|---|---|---|---|---|
| | **Control** | **Maternal** | **Paternal** | **Control** | **Maternal** | **Paternal** |
| No. genes | 20 242 | 20 | 15 | 16 877 | 30 | 26 |
| No. intronless genes | 1315 | $4^{0.045}$ | 0 | 2092 | $10^{0.001}$ | 0 |
| Total exon size | 2632 | 6829 | 2969 | 2277 | 8380 | 2558 |
| Total exon size[a] | 2692 | 4351 | 2969 | 2409 | 2292 | 2558 |
| Remove *SNRPN* | 2632 | 5970 | 2969 | 2277 | 8635 | 2558 |
| Remove *SNRPN*[a] | 2692 | 3098 | 2969 | 2409 | 2361 | 2558 |
| Average exon size | 429 | $3819^{0.036}$ | 333 | 467 | $7133^{0.014}$ | 322 |
| Average exon size[a] | 336 | 588 | 333 | 343 | 422 | 322 |
| Remove *SNRPN* | 429 | $4011^{0.016}$ | 333 | 467 | $7374^{0.006}$ | 322 |
| Remove *SNRPN*[a] | 336 | 617 | 333 | 343 | 436 | 322 |
| Total intron size | 53 268 | $41\,844^{0.039}$ | 74 132 | 37 926 | $20\,124^{0.003}$ | $53\,386^{0.01}$ |
| Total intron size[a] | 56 966 | 52 305 | 74 132 | 43 279 | 30 186 | 53 386 |
| Remove *SNRPN* | 53 268 | $23\,677^{0.012}$ | 74 132 | 37 926 | $20\,658^{0.004}$ | $53\,386^{0.01}$ |
| Remove *SNRPN*[a] | 56 966 | 29 990 | 74 132 | 43 279 | 31 530 | 53 386 |
| Average intron size | 6033 | 2801 | 6763 | 5177 | $2202^{0.002}$ | 5412 |
| Average intron size[a] | 6452 | 3501 | 6763 | 5904 | 3302 | 5412 |
| Remove *SNRPN* | 6033 | 2796 | 6763 | 5177 | $2251^{0.003}$ | 5412 |
| Remove *SNRPN*[a] | 6452 | 3542 | 6763 | 5904 | 3436 | 5412 |
| No. introns | 9.4 | 13.3 | 11.3 | 7.8 | $4.6^{0.004}$ | $10.3^{0.05}$ |
| No. introns[a] | 9.9 | 16.6 | 11.3 | 8.9 | 6.8 | 10.3 |
| Remove *SNRPN* | 9.4 | $6.2^{0.024}$ | 11.3 | 7.8 | $4.5^{0.003}$ | $10.3^{0.05}$ |
| Remove *SNRPN*[a] | 9.9 | 7.8 | 11.3 | 8.9 | 6.9 | 10.3 |
| Total intron/total exon size | 19.5 | $7.0^{0.006}$ | 19.8 | 16.3 | $6.3^{0.002}$ | 13.6 |
| Total intron/total exon size[a] | 20.8 | 8.7 | 19.8 | 18.6 | 9.4 | 13.6 |
| Remove *SNRPN* | 19.5 | $6.3^{0.003}$ | 19.8 | 16.3 | $6.7^{0.007}$ | 13.6 |
| Remove *SNRPN*[a] | 20.8 | $8.0^{0.023}$ | 19.8 | 18.6 | 9.6 | 13.6 |
| No. introns/kb exon | 3.6 | $2.5^{0.029}$ | 3.6 | 3.4 | 3.3 | $9.2^{0.000}$ |
| No. introns/kb exon[a] | 3.9 | $3.2^{0.045}$ | 3.6 | 3.9 | 4.9 | $9.2^{0.001}$ |
| Remove *SNRPN* | 3.6 | $2.3^{0.01}$ | 3.6 | 3.4 | $6.3^{0.002}$ | $9.2^{0.000}$ |
| Remove *SNRPN*[a] | 3.9 | 2.9 | 3.6 | 3.9 | 4.8 | $9.2^{0.001}$ |

The average values for each parameter are shown for both human and mouse imprinted and non-imprinted genes. Non-protein coding imprinted genes are included in this analysis.

[a] Indicates analyses from which intronless genes were excluded. *SNRPN* was removed from the analysis, alone and in combination with intronless genes. Average intron sizes and average exon sizes were calculated based on the average of averages for each gene within a dataset. Imprinted genes are divided into those silenced on either the maternally or paternally inherited chromosome, and a Mann–Whitney U Test was employed to test the null hypothesis that imprinted and non-imprinted genes are similar with respect to gene structure. A Fisher's exact test was used to compare the number of intronless genes observed in the groups.

Intron content of a gene is influenced both by intron size and intron number. Can we specify whether our observations of reduced and increased intron content, respectively, of maternally and paternally silenced genes is due to effects on intron size, intron number, or both? Comparison of the maternally or paternally silenced genes with the control dataset detected differences in both of these parameters in the mouse and human, but the differences were not always statistically significant in both species. Indeed, there is evidence for species-specific effects because (following removal of *SNRPN*), intron number in maternally silenced genes is reduced in both human and mouse, whereas in the paternally silenced datasets, intron number is increased in mouse but not in human. In both species, average intron length is reduced in the maternally silenced datasets; however, the difference is significant only in the mouse, and depends on the inclusion of intronless genes.

Copyright © 2005 John Wiley & Sons, Ltd. 

**Table 3.** Genomic structure of maternally and paternally silenced imprinted genes excluding non-protein coding genes

| | Human | | | Mouse | | |
|---|---|---|---|---|---|---|
| | Control | Maternal | Paternal | Control | Maternal | Paternal |
| No. genes | 20 242 | 19 | 14 | 16 877 | 26 | 24 |
| No. intronless genes | 1315 | $3^{0.035}$ | 0 | 2085 | $8^{0.01}$ | 0 |
| Total exon size | 2632 | 4031 | 3016 | 2277 | 2338 | 2622 |
| Total exon size[a] | 2692 | 4351 | 3016 | 2409 | 2220 | 2622 |
| Remove *SNRPN* | 2632 | 2969 | 3016 | 2277 | 2391 | 2622 |
| Remove *SNRPN*[a] | 2692 | 3098 | 3016 | 2409 | 2292 | 2622 |
| Average exon size | 429 | 862 | 324 | 467 | 1051 | 323 |
| Average exon size[a] | 336 | 588 | 324 | 343 | 361 | 323 |
| Remove *SNRPN* | 429 | $901^{0.039}$ | 324 | 467 | 1087 | 323 |
| Remove *SNRPN*[a] | 336 | 617 | 324 | 343 | 374 | 323 |
| Total intron size | 53 268 | 44 046 | 79 402 | 37 926 | $22\,754^{0.016}$ | $57\,272^{0.004}$ |
| Total intron size[a] | 56 966 | 52 305 | 79 402 | 43 279 | 32 867 | $57\,272^{0.033}$ |
| Remove *SNRPN* | 53 268 | $24\,992^{0.029}$ | 79 402 | 37 926 | $23\,478^{0.021}$ | $57\,272^{0.004}$ |
| Remove *SNRPN*[a] | 56 966 | 29 990 | 79 402 | 43 279 | 34 527 | $57\,272^{0.033}$ |
| Average intron size | 6033 | 2948 | 7241 | 5177 | $2330^{0.004}$ | $5781^{0.033}$ |
| Average intron size[a] | 6452 | 3501 | 7241 | 5904 | 3365 | 5781 |
| Remove *SNRPN* | 6033 | 2952 | 7241 | 5177 | $2392^{0.006}$ | $5781^{0.033}$ |
| Remove *SNRPN*[a] | 6452 | 3542 | 7241 | 5904 | 3518 | 5781 |
| No. introns | 9.4 | 14.0 | 11.8 | 7.8 | $5.1^{0.032}$ | $10.7^{0.042}$ |
| No. introns[a] | 10.0 | 16.6 | 11.8 | 8.9 | 7.4 | 10.7 |
| Remove *SNRPN* | 9.4 | 6.5 | 11.8 | 7.8 | $5.1^{0.028}$ | $10.7^{0.042}$ |
| Remove *SNRPN*[a] | 10.0 | 7.8 | 11.8 | 8.9 | 7.4 | 10.7 |
| Total intron/total exon size | 19.5 | $7.3^{0.015}$ | 21.2 | 16.3 | $6.8^{0.01}$ | 14.5 |
| Total intron/total exon size[a] | 20.8 | $8.7^{0.045}$ | 21.2 | 18.6 | 9.9 | 14.5 |
| Remove *SNRPN* | 19.5 | $6.6^{0.007}$ | 21.2 | 16.3 | $6.9^{0.01}$ | 14.5 |
| Remove *SNRPN*[a] | 20.8 | $7.9^{0.023}$ | 21.2 | 18.6 | 10.2 | 14.5 |
| No. introns/kb exon | 3.6 | 2.7 | 3.7 | 3.4 | 3.6 | $9.5^{0.000}$ |
| No. introns/kb exon[a] | 3.9 | 3.2 | 3.7 | 3.9 | 5.3 | $9.5^{0.001}$ |
| Remove *SNRPN* | 3.6 | $2.4^{0.023}$ | 3.7 | 3.4 | 3.5 | $9.5^{0.000}$ |
| Remove *SNRPN*[a] | 3.9 | 2.9 | 3.7 | 3.9 | 5.2 | $9.5^{0.001}$ |

Data reported as in Table 2. Non-protein coding genes excluded from the analysis, human *LIT1* and *H19*, and mouse *Igf2as, Peg13, Mirg* and *H19*.

Therefore, we cannot determine unambiguously, from the current datasets, the source of altered intron content of imprinted genes relative to controls. However, the data in Table 4 which, unlike those in Tables 2 and 3, are not compiled on a 'per gene' basis, show more clearly that (following exclusion of intronless genes and *SNRPN*) there are systematic differences across species between maternally and paternally silenced genes for both intron number and intron size.

Direct comparison of mouse maternally and paternally silenced datasets detected significant differences between them for a range of intron-related parameters. A similar analysis of human imprinted genes, however, did not. This may be due to the lower number of imprinted genes in our Modified Human Imprinted Gene List. Trends in the data are observed more clearly in Table 4. A marked reduction is observed in both intron number and intron length, in both species and across all parameters. These differences may achieve statistical significance in future studies, subject to the discovery or full structural characterization of further imprinted genes. For example, total intron size of the human paternally silenced dataset is larger than that of the maternally silenced and control datasets, but the differences may not have reached significance because of the relatively small number (14) of

Copyright © 2005 John Wiley & Sons, Ltd. 

**Table 4.** Observed trends in human and mouse datasets

| | | | | | |
|---|---|---|---|---|---|
| Number of introns | | | | | |
| Human | Pat(11.8) | > | Control(10) | > | Mat(7.8) |
| Mouse | Pat(10.7) | > | Control(8.9) | > | Mat(7.4) |
| Total intron size | | | | | |
| Human | Pat(79 402) | > | Control(56 966) | > | Mat(29 990) |
| Mouse | Pat(57 272) | > | Control(43 279) | > | Mat(34 527) |
| Total exon size | | | | | |
| Human | Pat(3016) | | Control(2692) | | Mat(3098) |
| Mouse | Pat(2622) | > | Control(2409) | > | Mat(2292) |
| Average intron size | | | | | |
| Human | Pat(6717) | > | Control(5780) | > | Mat(3824) |
| Mouse | Pat(5362) | > | Control(4905) | > | Mat(4646) |
| Average exon size | | | | | |
| Human | Mat(350) | > | Control(253) | > | Pat(235) |
| Mouse | Mat(272) | > | Control(248) | > | Pat(224) |
| Total intron size/total exon size | | | | | |
| Human | Pat(26.3) | > | Control(21) | | Mat(9.7) |
| Mouse | Pat(21.8) | > | Control(18) | > | Mat(15.1) |
| No. introns/kb exon | | | | | |
| Human | Pat(3.9) | > | Control(3.7) | > | Mat(2.5) |
| Mouse | Pat(4.1) | > | Control(3.7) | > | Mat(3.2) |

Average value for each parameter is reported. Data excludes *SNRPN*, intronless and non-coding genes. Average intron length and average exon length are not averages of averages but one average taken from all individual introns and exons in each group. Total intron length/total exon length is calculated from the two average values reported for both parameters previously. Number of introns per kilobase of exon is a ratio of number of introns and total exon length. Consistent trends of reduced intron content (both size and number) of maternally silenced genes are observed in both species.

**Table 5.** Conservation of imprinted intronless genes between mouse and human

| | **Human** | | **Mouse** | |
|---|---|---|---|---|
| | **Imprinted** | **Intronless** | **Imprinted** | **Intronless** |
| *Mkrn3* | + | + | + | + |
| *Ndn* | + | + | + | + |
| *Magel2* | + | + | + | + |
| *Lit1* | + | + | + | * |
| *Frat3* | − | − | + | + |
| *U2af1-rs1* | − | + | + | + |
| *Dio3* | * | + | + | + |
| *Rtl1*[a] | * | * | + | + |
| *Peg13* | * | * | + | + |
| *Air* | − | − | + | + |
| *Nap1l5* | − | + | + | + |

+, Imprinted/intronless; −, not conserved. * Insufficient data.
[a] A model *Rtl1* transcript maps to chromosome 14q32.32 (RefSeq XM_352 144). Human *U2AF1-RS1* is an intronless gene mapping to chromosome 5, but appears to have a separate evolutionary origin (retrotransposition) to mouse *U2af1-rs1*, so the genes are non-orthologous.

**Table 6.** Comparison of mouse maternally and paternally silenced genes

| **Parameters** | **Significant *p*-values** |
|---|---|
| No. intronless genes | 0.005 |
| Total exon size | — |
| Average exon size | — |
| Total intron size | 0.001 |
| Average intron size | 0.001 |
| No. introns | 0.031 |
| Total intron size/total exon size | — |
| No. introns/kb exon | 0.003 |

Datasets include *SNRPN* and intronless genes, but exclude non-coding genes. Parameters showing no significant differences are denoted by dashes. Those parameters which are significantly different are unaffected by either the removal of *SNRPN* or by the inclusion of non-coding genes. However, exclusion of intronless genes abolishes significance for all parameters.

well-characterised human paternally silenced genes available.

The choice of control and imprinted genes for inclusion in our datasets is not trivial. Known imprinted genes were removed from the RefSeq databases, but these datasets may nevertheless contain unidentified imprinted genes. However, the number of imprinted genes in the genome is probably quite small (Moore, 2001), and unlikely to significantly bias the large control datasets used in this study. Moreover, any such bias would tend to produce a false negative, but not a false positive, result, i.e. it would increase the similarity between the imprinted and control datasets. The selection of imprinted gene datasets is also problematic because, given the relatively small number of imprinted genes, the inclusion or exclusion of a single gene might have a significant effect on the mean parameter values of the dataset. For example, the maternally silenced *SNRPN* gene has a significant effect on several parameters because it contains a large number of introns that encode SnoRNAs. This gene is also problematical because the mouse orthologue is not fully characterised. We therefore re-analysed all parameters following the exclusion of *SNRPN*, and find that while it has a significant effect on some parameters in the human, the observation of reduced intron content of maternally silenced genes compared to the control set is retained irrespective of *SNRPN* inclusion or exclusion. The inclusion of a single non-coding gene in the human maternally silenced dataset, with exclusion of *SNRPN*, results in a further two

Copyright © 2005 John Wiley & Sons, Ltd. 

parameters becoming significantly different from controls: total intron size and the number of introns per kilobase of exon. As the human imprinted data set becomes more complete, these findings may become more robust. Mouse data in general are less sensitive to the exclusion or inclusion of one or more genes.

In addition, there are a variety of methods of determining whether a gene is subject to imprinting, not all of which lead to similar conclusions. Therefore, the imprinted status of some genes is controversial. Our datasets contain three such genes (human *GRB10, IGF2R, COPG2*); however, their removal does not affect our conclusions.

We set out to confirm the previously reported finding that imprinted genes are unusual with respect to intron content compared to non-imprinted genes (Hurst *et al.*, 1996). Initially, in the present study, we found that neither human nor mouse datasets comprising both maternally and paternally silenced imprinted genes were different to our control gene datasets. Because the imprinted gene dataset used in the original study of Hurst *et al.* (1996) was small and somewhat biased towards maternally silenced genes (9 out of 14; not including mouse *Mas* and human *CG*, which are now thought not to be imprinted), we hypothesised that the observation of reduced intron content of imprinted genes was due primarily to the relatively high maternally silenced gene content. We therefore analysed maternally and paternally silenced genes separately. We applied a Bonferroni correction at an $\alpha$-level of 0.012 instead of 0.05 to control for these two separate tests and also to account for testing for multiple parameters (intron size and intron number) (Pernager, 1998). We note that in the direct comparison of mouse maternally and paternally silenced gene datasets, five of eight parameters would withstand a severe Bonferroni correction.

We considered and excluded a number of factors that might, in principle, explain our observations. Castillo-Davis *et al.* (2002) showed that, in the human and in *C. elegans*, introns of highly expressed genes are 14-fold and two-fold shorter, respectively, than in genes with low expression. However, they detected no difference in intron density. However, our observation of reduced intron size in maternally silenced genes of the mouse, with a similar trend in the human, is not explained by differences in imprinted gene expression levels, because EST counts were similar for both maternally and paternally silenced gene datasets.

There are complex, and probably overlapping, effects of a variety of parameters, such as GC and transposon content, and genetic recombination rate, on intron content (Hurst *et al.*, 1999; Duret, 2001). Human maternally and paternally imprinted gene regions differ in GC and transposon content (Greally, 2002), and some imprinted regions exhibit higher levels of recombination in the male, relative to the female, germline (Paldi *et al.*, 1995; Robinson and Lalande, 1995). However, none of these parameters appears to provide a convincing explanation for our observations. For example, the paternally silenced subgroup has a relatively high GC content (Greally, 2002), which is an expected correlate of short introns, contrary to our finding of a trend towards reduced intron length in both mouse and human maternally silenced datasets. Moreover, we found no correlation between recombination rate and intron content in the human imprinted gene dataset. Indeed, inspection of Table 1 indicates that, contrary to expectation, among imprinted genes, intronless genes map to regions of relatively low recombination. An additional, recent analysis of recombination in imprinted regions found that imprinted regions have high recombination rates compared to non-imprinted regions of the genome; however, there was no evidence that maternally and paternally silenced imprinted genes are different with respect to local recombination rate (Lercher and Hurst, 2003).

We could not explain our finding of reduced intron content of maternally silenced genes in terms of gene function or genomic location, therefore we considered population genetic arguments that might, in principle, explain our observations. Lynch (2002) has proposed that the phylogenetic distribution of intron density may be explained by considering introns as weakly deleterious, and therefore subject to purifying selection or random genetic drift, depending on species population size. However, weak purifying selection against introns may be countered by genetic hitch-hiking. The rate at which a linked, beneficial mutation approaches fixation during a selective sweep would influence the probability of recombination between an intron-containing allele and an intronless variant. We expect rapidly evolving loci to fix introns more frequently than relatively slowly evolving

Copyright © 2005 John Wiley & Sons, Ltd. 

loci, because there is less chance of recombination with an intronless variant during a selective sweep and less time for purifying selection to select between intronless and intron-containing variants. From these arguments, we tentatively propose that the possibility that there are systematically different rates of evolution of maternally and paternally silenced imprinted genes (Mills and Moore, 2004) may provide a explanation for our observations.

### Methods

### Gene structure analysis

Mouse and human control gene datasets were obtained from the UCSC genome site (**http://genome.ucsc.edu**) (October 2003) and contained 20 248 and 16 883 full-length human and mouse transcript sequences, respectively, from the Refseq database (Pruitt and Maglott, 2001). Tables outlining the gene structure of the transcripts are available from an alignment of the mRNA sequence to the human draft sequence of June 2003 and mouse of February 2003. The RefSeq database is a curated, non-redundant database at the NCBI consisting of full-length sequences as currently described. The database aims to have one reference sequence for each transcript in the genome. Our control sets therefore represent a global, unbiased sample of mouse and human genes.

A list of mouse and human imprinted genes was obtained from the MRC Mammalian Genetics Unit (**http://www.mgu.har.mrc.ac.uk**) and the Catalogue of Imprinted Genes and Parent-of-Origin Effects databases (Morison *et al*., 2001), respectively, and was supplemented by searching for new imprinted genes in PubMed. Imprinted genes were removed from the Refseq control datasets. Maternally and paternally silenced gene datasets were compared to non-imprinted genes using a non-parametric statistical test. The mean value of each parameter was used and, in cases where different transcript variants exist for an imprinted gene, the average values were calculated for each transcript and subsequently an overall average was taken. Analyses were carried out including and excluding intronless genes, genes whose imprinted status is controversial, and the *SNURF-SNRPN* snoRNA-containing transcript (Runte *et al*., 2001). A chi- squared test with Yates' correction was used to ascertain the significance of the number of imprinted genes without introns compared to the control gene dataset. Conservation of imprinting between mouse and human orthologues was also investigated. In most cases orthologue pairs are annotated. However, in cases where one is missing, sequence identity in the corresponding genome was searched using the BLAT program at UCSC, which is designed to find regions of high sequence identity.

*GNAS* is a highly complex locus with respect to both transcript-specific transcription and imprinted expression patterns. The locus maps to chromosome 20 in the human and chromosome 2 in the mouse. It is composed of both sense and antisense transcripts, which are associated with alternative first exons. *Nesp* is a paternally silenced gene; *Gnasxl, Nespas* and *Gnas exon 1a* are maternally silenced; *Gnas exon 1* is expressed from both alleles; however, there is evidence for tissue specific imprinting (Yu *et al*., 1998; Liu *et al*., 2003). Holmes *et al*. (2003) further characterized this locus using the FANTOM mouse transcriptome (Okazaki *et al*., 2002) and identified new alternative transcripts, which they labelled F1–F12. Both spliced and unspliced variants were published with alternative 3′ untranslated regions. Using the FANTOM database (**http://fantom.gsc.riken.go.jp/db**) we took the genomic structure for each variant and averaged transcript length for *Gnas exon 1a, Nesp* and *Gnasxl* (clones D930047C10 and A930027G11; A230089C09 and D930020N02; C130027O20 and 533 041BM12).

### Gene expression analysis

Expression levels of imprinted genes were estimated from expressed sequence tag (EST) abundance in the public databases. BLASTN (version 2.2.4) was used to compare imprinted gene transcript sequences to a database of 4 533 427 human EST sequences downloaded from NCBI [31] (August 2002). Threshold values were set to allow EST hits of $>400$ nucleotides with $>95\%$ identity to be accepted as matches. If identity exceeded 98%, sequence alignment of 100–400 nucleotides was also accepted. Non-coding genes and *SNRPN* were excluded from the analysis.

Copyright © 2005 John Wiley & Sons, Ltd.

### Recombination rate

Human genetic recombination rates on the deCODE genetic map [32] were obtained from the UCSC Genome Browser (**http://genome.ucsc.edu**).

### Authors' contributions

MF participated in the design of the study, carried out bioinformatic and data analyses, and drafted the manuscript. W.M., D.H. and T.M. participated in the design of the study and drafted the manuscript. All authors read and approved the final manuscript.

### Acknowledgements

We acknowledge support from the Biopharmaceutical Sciences Network, funded by the Irish Higher Education Authority, and from the Health Research Board. T.M. is an Irish Health Research Board/Wellcome Trust 'New Blood' Research Fellow. We thank F. Kondrashov for supplying EST expression data used in Castillo-Davis *et al.* (2002), P. McKeigue for advice on statistical analysis, and anonymous reviewers for helpful comments.

### References

Blagitko N, Mergenthaler S, Schulz U, *et al.* 2000. Human GRB10 is imprinted and expressed from the paternal and maternal allele in a highly tissue- and isoform-specific fashion. *Hum Mol Genet* **9**(11): 1587–1595.

Carvalho AV, Clark AG. 1999. Genetic recombination: intron size and natural selection. *Nature* **401**: 344.

Castillo-Davis CI, Mekhedov SL, Hartl DL, Koonin EV, Kondrashov FA. 2002. Selection for short introns in highly expressed genes. *Nature Genet* **31**(4): 415–418.

Fantom database (Functional Annotation of Mouse); **http://fantom.gsc.riken.go.jp/db**

Chai JH, Locke DP, Ohta T, Greally JM, Nicholls RD. 2001. Retrotransposed genes such as *Frat3* in the mouse chromosome 7C Prader–Willi syndrome region acquire the imprinted status of their insertion site. *Mamm Genome* **12**(11): 813–821.

Deutsch M, Long M. 1999. Intron-exon structures of eukaryotic model organisms. *Nucleic Acid Res* **27**(15): 3219–3228.

Duret L, Mouchiroud D, Gautier C. 1995. Statistical analysis of vertebrate sequences reveals that long genes are scarce in GC-rich isochors. *J Mol Evol* **40**: 308–317.

Duret L. 2001. Why do genes have introns? Recombination might add a new piece to the puzzle. *Trends Genet* **17**(4): 172–175.

Greally JM. 2002. Short interspersed transposable elements (SINEs) are excluded from imprinted regions in the human genome. *Proc Natl Acad Sci USA* **99**(1): 327–332.

Holmes R, Williamson C, Peters J, Denny P, RIKEN GER Group and GSL members, Wells C. 2003. A comprehensive map transcript map of the mouse Gnas imprinted locus. *Genome Res* **13**: 1410–1415.

Hurst LD, Brunton CFA, Smith NGC. 1999. Small introns tend to occur in GC-rich regions in some but not all vertebrates. *Trends Genet* **15**(11): 437–439.

Hurst LD, McVean G, Moore T. 1996. Imprinted genes have few and small introns. *Nature Genet* **12**(3): 234–237.

John RM, Surani MA. 1996. Imprinted genes and regulation of gene expression by epigenetic inheritance. *Curr Opin Cell Biol* **8**(3): 348–353.

Kalscheuer VM, Mariman EC, Schepens MT, Rehder H, Ropers HH. 1993. The insulin-like growth factor type-2 receptor gene is imprinted in the mouse but not in humans. *Nature Genet* **5**(1): 74–78.

Kong A, Gudbjartsson DF, Sainz J, *et al.* 2002. A high resolution recombination map of the human genome. *Nature Genet* **31**(3): 241–247.

Lercher MJ, Hurst LD. 2003. Imprinted chromosomal regions of the human genome have unusually high recombination rates. *Genetics* **165**: 1629–1632.

Liu J, Erlichman B, Weinstein LS. 2003. The stimulatory G protein $\alpha$-subunit Gs-$\alpha$ is imprinted in human thyroid glands: implications for thyroid function in pseudohypoparathyroidism types 1A and 1B. *J Clin Endocrinol Metab* **88**(9): 4336–4341.

Lynch M. 2002. Intron evolution as a population-genetic process. *Proc Natl Acad Sci USA* **99**(9): 6118–6123.

Mammalian Genetics Unit **http://www.mgu.har.mrc.ac.uk/research/imprinted**

Mills W, Moore T. 2004. Polyandry, life-history trade-offs and the evolution of imprinting at Mendelain loci. *Genetics* (in press).

Moore T, Haig D. 1991. Genomic imprinting in mammalian development: a parental tug-of-war. *Trends Genet* **7**(2): 45–49.

Morison IM, Paton CJ, Cleverley SD. 2001. The imprinted gene and parent-of-origin effect database. *Nucleic Acids Research* **29**(1): 275–276; **http://cancer.otago.ac.nz/IGC/Web/home.html**

Nabetani A, Hatada I, Morisaki H, Oshimura M, Mukai T. 1997. Mouse U2af1-rs1 is a neomorphic imprinted gene. *Mol Cell Biol* **17**(2): 789–798.

NCBI FTP server; **ftp://ftp.ncbi.nih.gov/blast/db**

Okazaki Y, Furuno M, Kasukawa T, *et al.* 2002. Analysis of the mouse transcriptome based on functional annotation of 60 770 full-length cDNAs. *Nature* **420**(6915): 563–573.

Okutsu T, Kuroiwa Y, Kagitani F, *et al.* 2000. Expression and imprinting status of human PEG8/IGF2AS, a paternally expressed antisense transcript from the *IGF2* locus, in Wilms' tumors. *J Biochem* **127**(3): 475–483.

Paldi A, Jami J, Gyapay G, Paldi A. 1995. Imprinted chromosomal regions of the human genome display sex-specific meiotic recombination frequencies. *Curr Biol* **5**(9): 1030–1035.

Pernager TV. 1998. What is wrong with Bonferroni adjustments. *Br. Med J* **136**: 1236–1238.

Pruitt K, Maglott D. 2001. RefSeq and LocusLink: NCBI gene-centered resources. *Nucleic Acid Res* **29**(1): 137–140.

Robinson WP, Lalande M. 1995. Sex-specific meiotic recombination in the Prader–Willi/Angelman syndrome imprinted region. *Hum Mol Genet* **4**(5): 801–806.

Rogozin IB, Wolf YI, Sorokin AV, Mirkin BG, Koonin EV. 2003. Remarkable interkingdom conservation of intron positions and massive, lineage-specific intron loss and gain in eukaryotic evolution. *Curr Biol* **13**(17): 1512–1517.

Runte M, Huttenhofer A, Gross S, *et al.* 2001. The IC-SNURF_SNRPN transcript serves as a host for multiple small

Copyright © 2005 John Wiley & Sons, Ltd. 

nucleolar RNA species and as an antisense RNA for UBE3A. *Hum Mol Gen* **10**(23): 2687–2700.

UCSC Genome Bioinformatics; **http://genome.ucsc.edu**

Williamson CM, Skinner JA, Kelsey G, Peters J. 2002. Alternative non-coding splice variants of *Nespas*, an imprinted gene antisense to *Nesp* in the *Gnas* imprinting cluster. *Mamm Genome* **13**: 74–79.

Yamasaki K, Hayashida S, Miura K, *et al*. 2000. The novel gene, $\gamma$2-COP (COPG2), in the 7q32 imprinted domain escapes genomic imprinting. *Genomics* **68**(3): 330–335.

Copyright © 2005 John Wiley & Sons, Ltd. 

## References

[PDF_00987] Blagitko N., Mergenthaler S., Schulz U., Wollmann H. A., Craigen W., Eggermann T., Ropers H. H., Kalscheuer V. M. (2000). Human GRB10 is imprinted and expressed from the paternal and maternal allele in a highly tissue- and isoform-specific fashion.. Hum Mol Genet.

[PDF_00991] Carvalho A. B., Clark A. G. (1999). Intron size and natural selection.. Nature.

[PDF_00993] Castillo-Davis Cristian I., Mekhedov Sergei L., Hartl Daniel L., Koonin Eugene V., Kondrashov Fyodor A. (2002). Selection for short introns in highly expressed genes.. Nat Genet.

[PDF_00998] Chai J. H., Locke D. P., Ohta T., Greally J. M., Nicholls R. D. (2001). Retrotransposed genes such as Frat3 in the mouse Chromosome 7C Prader-Willi syndrome region acquire the imprinted status of their insertion site.. Mamm Genome.

[PDF_01002] Deutsch M., Long M. (1999). Intron-exon structures of eukaryotic model organisms.. Nucleic Acids Res.

[PDF_01004] Duret L., Mouchiroud D., Gautier C. (1995). Statistical analysis of vertebrate sequences reveals that long genes are scarce in GC-rich isochores.. J Mol Evol.

[PDF_01007] Duret L. (2001). Why do genes have introns? Recombination might add a new piece to the puzzle.. Trends Genet.

[PDF_01009] Greally John M. (2001). Short interspersed transposable elements (SINEs) are excluded from imprinted regions in the human genome.. Proc Natl Acad Sci U S A.

[PDF_01012] Holmes Rebecca, Williamson Christine, Peters Jo, Denny Paul, Wells Christine, RIKEN GER Group, GSL Members (2003). A comprehensive transcript map of the mouse Gnas imprinted complex.. Genome Res.

[PDF_01016] Hurst L. D., Brunton C. F., Smith N. G. (1999). Small introns tend to occur in GC-rich regions in some but not all vertebrates.. Trends Genet.

[PDF_01019] Hurst L. D., McVean G., Moore T. (1996). Imprinted genes have few and small introns.. Nat Genet.

[PDF_01021] John R. M., Surani M. A. (1996). Imprinted genes and regulation of gene expression by epigenetic inheritance.. Curr Opin Cell Biol.

[PDF_01024] Kalscheuer V. M., Mariman E. C., Schepens M. T., Rehder H., Ropers H. H. (1993). The insulin-like growth factor type-2 receptor gene is imprinted in the mouse but not in humans.. Nat Genet.

[PDF_01028] Kong Augustine, Gudbjartsson Daniel F., Sainz Jesus, Jonsdottir Gudrun M., Gudjonsson Sigurjon A., Richardsson Bjorgvin, Sigurdardottir Sigrun, Barnard John, Hallbeck Bjorn, Masson Gisli (2002). A high-resolution recombination map of the human genome.. Nat Genet.

[PDF_01031] Lercher Martin J., Hurst Laurence D. (2003). Imprinted chromosomal regions of the human genome have unusually high recombination rates.. Genetics.

[PDF_01034] Liu Jie, Erlichman Beth, Weinstein Lee S. (2003). The stimulatory G protein alpha-subunit Gs alpha is imprinted in human thyroid glands: implications for thyroid function in pseudohypoparathyroidism types 1A and 1B.. J Clin Endocrinol Metab.

[PDF_01038] Lynch Michael (2002). Intron evolution as a population-genetic process.. Proc Natl Acad Sci U S A.

[PDF_01044] Moore T., Haig D. (1991). Genomic imprinting in mammalian development: a parental tug-of-war.. Trends Genet.

[PDF_01046] Morison I. M., Paton C. J., Cleverley S. D. (2001). The imprinted gene and parent-of-origin effect database.. Nucleic Acids Res.

[PDF_01050] Nabetani A., Hatada I., Morisaki H., Oshimura M., Mukai T. (1997). Mouse U2af1-rs1 is a neomorphic imprinted gene.. Mol Cell Biol.

[PDF_01054] Okazaki Y., Furuno M., Kasukawa T., Adachi J., Bono H., Kondo S., Nikaido I., Osato N., Saito R., Suzuki H. (2002). Analysis of the mouse transcriptome based on functional annotation of 60,770 full-length cDNAs.. Nature.

[PDF_01057] Okutsu T., Kuroiwa Y., Kagitani F., Kai M., Aisaka K., Tsutsumi O., Kaneko Y., Yokomori K., Surani M. A., Kohda T. (2000). Expression and imprinting status of human PEG8/IGF2AS, a paternally expressed antisense transcript from the IGF2 locus, in Wilms' tumors.. J Biochem.

[PDF_01066] Pruitt K. D., Maglott D. R. (2001). RefSeq and LocusLink: NCBI gene-centered resources.. Nucleic Acids Res.

[PDF_01061] Pàldi A., Gyapay G., Jami J. (1995). Imprinted chromosomal regions of the human genome display sex-specific meiotic recombination frequencies.. Curr Biol.

[PDF_01068] Robinson W. P., Lalande M. (1995). Sex-specific meiotic recombination in the Prader--Willi/Angelman syndrome imprinted region.. Hum Mol Genet.

[PDF_01071] Rogozin Igor B., Wolf Yuri I., Sorokin Alexander V., Mirkin Boris G., Koonin Eugene V. (2003). Remarkable interkingdom conservation of intron positions and massive, lineage-specific intron loss and gain in eukaryotic evolution.. Curr Biol.

[PDF_01082] Williamson Christine M., Skinner Judith A., Kelsey Gavin, Peters Josephine (2002). Alternative non-coding splice variants of Nespas, an imprinted gene antisense to Nesp in the Gnas imprinting cluster.. Mamm Genome.

[PDF_01086] Yamasaki K., Hayashida S., Miura K., Masuzaki H., Ishimaru T., Niikawa N., Kishino T. (2000). The novel gene, gamma2-COP (COPG2), in the 7q32 imprinted domain escapes genomic imprinting.. Genomics.

